# Cutaneous endothelial cells and their roles in wound healing

**DOI:** 10.1016/j.xjidi.2026.100539

**Published:** 2026-09-10

**Authors:** Haiming Li, Laura Sormani Le Bourhis, Seen-Ling Sim, Abbas Shafiee, Kiarash Khosrotehrani

**Affiliations:** 1 Frazer Institute, Faculty of Health, Medicine and Behavioural Sciences, The University of Queensland, Brisbane, Queensland, Australia; 2 Metro North Hospital and Health Service, Queensland Health, Brisbane, Queensland, Australia; 3 Department of Dermatology, Princess Alexandra Hospital, Brisbane, Queensland, Australia

**Keywords:** Angiogenesis, Inflammation, Regenerative medicine, Scarring, Vascular–immune crosstalk

## Abstract

Endothelial cells (ECs) form the inner monolayer of blood and lymphatic vessels play an essential role in skin homeostasis and wound healing. Upon injury, ECs rapidly engage in the healing response. ECs promote wound healing by forming new blood vessels through angiogenesis to supply oxygen, nutrients, and signals needed for tissue repair. Diseases like diabetes can cause endothelial dysfunction, leading to defective neovascularization and delayed wound closure. This review focuses on the intrinsic roles of ECs in each phase of skin wound healing, extending beyond their roles in vascularization, and discusses therapeutic strategies that target those functions to improve healing outcomes. Recognizing ECs as dynamic regulators of wound repair, rather than merely structural components of blood vessels, could open new therapeutic avenues for regenerative skin therapies.

## Introduction

The skin is the largest organ by surface area and weight in the human body. Effective cutaneous wound healing is essential to restore skin integrity after injury as it is a highly conserved evolutionary process that has major implications for a variety of diseases. In homeostasis, dermal endothelial cells (ECs) maintain vascular integrity, regulate blood flow and permeability via antithrombotic factors (eg, nitric oxide) and support immune surveillance (Krüger-Genge et al, 2019; Rodrigues et al, 2019). Although dermal ECs remain quiescent during skin homeostasis, they are activated by hypoxia and inflammatory cytokines after injury and play critical roles in every phase of wound healing (Lallo and Bracaglia, 2024). Following skin injury, 4 sequential but also overlapping phases of wound healing (hemostasis, inflammation, proliferation, and remodeling) are initiated to restore tissue integrity (Rodrigues et al, 2019).

When skin homeostasis is disturbed, ECs regulate the transition from physiological maintenance to the tissue repair phase by forming new blood vessels, increasing vascular permeability, directing immune cell recruitment, and secreting growth factors that promote tissue regeneration (Peña and Martin, 2024). Disruption of the endothelium lining exposes perivascular collagen and tissue factors, which trigger platelet adhesion and activate fibrin clot formation (Olgasi et al, 2024). Subsequently, ECs orchestrate leukocyte recruitment during the inflammatory phase of wound healing through expression of adhesion molecules and chemokines or cytokines (Zhang et al, 2021). Activated ECs recruit neutrophils and monocytes and transiently loosen cell–cell junctions to facilitate diapedesis (Rho et al, 2017; Schulte et al, 2011) (Figure 1). During the proliferation phase of wound healing, ECs proliferate and migrate to form new capillaries through angiogenesis within the collagen-rich granulation tissue (Wallace et al, 2025). Finally, capillary density is selectively reduced, and the vascular pattern is restored during the remodeling phase. Thus, impairments in endothelial biology can severely delay effective wound healing (Han et al, 2022). Understanding EC roles at a mechanistic level has been an active research focus because it offers new therapeutic avenues. This review covers recent advances in skin EC biology, their roles in each phase of wound healing extending beyond their roles in vascularization, the impact of EC dysfunction in disease (especially diabetes and aging), and emerging therapeutic strategies that target ECs to improve healing outcomes.

**Figure 1:**
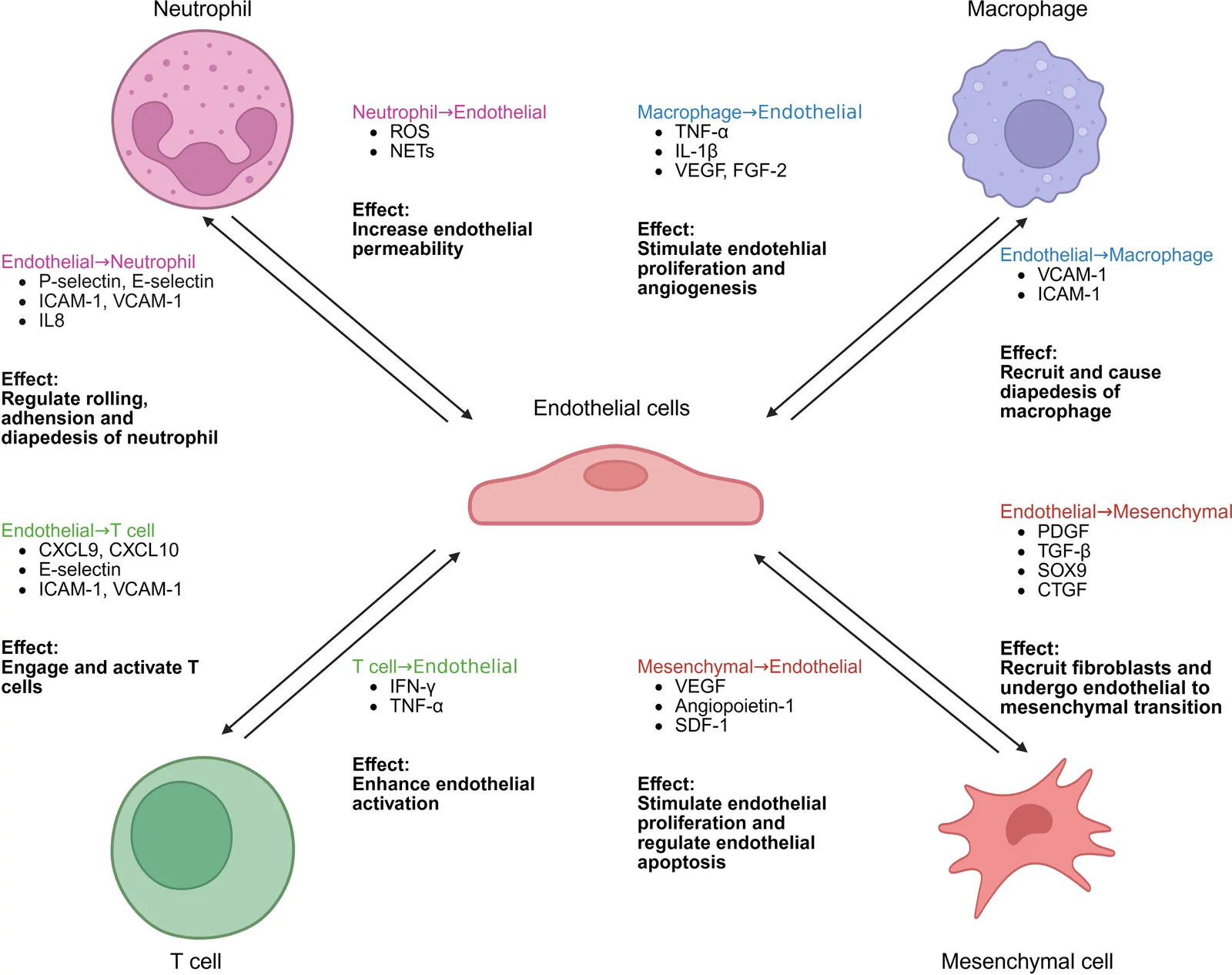
**Endothelial crosstalk in skin repair.** Schematic interactions between endothelial cells, and other cells involved in cutaneous wound healing processes. Created in BioRender. Li (2026) https://BioRender.com/di49skb. CTGF, connective tissue growth factor; CXCL9, C-X-C motif chemokine ligand 9; CXCL910, C-X-C motif chemokine ligand 10; FGF-2, fibroblast growth factor 2; NETs, neutrophil extracellular traps; PDGF, platelet-derived growth factor; SDF-1, stromal cell-derived factor-1; SOX9, SRY-box transcription factor 9; VCAM-1, vascular cell adhesion molecule-1.

## Cutaneous ECs

The cutaneous microvasculature is arranged in 2 horizontal plexuses: a superficial papillary plexus (around 1 to 1.5 mm below the epidermis) and a deeper reticular plexus (Braverman, 2000). A single layer of ECs lines the inner layer of all blood vessels. These cells have polarized basal and luminal surfaces and sit on a basement membrane. Each EC is connected to its neighbors by junctional proteins (eg, VE-cadherin) linking to the actin cytoskeleton (Giannotta et al, 2013).

Under normal conditions, skin ECs actively maintain homeostasis. They produce vasodilators (eg, nitric oxide) and vasoconstrictors (eg, endothelin) to adjust tone and secrete anticoagulants (eg, thrombomodulin) to inhibit clotting (Krüger-Genge et al, 2019). ECs also express anticoagulants (eg, heparan sulfate proteoglycans) that limit coagulation and complement activation (Pober and Sessa, 2007). ECs constantly regulate permeability via junctional remodeling. Such mechanisms allow plasma fluid and proteins to reach tissues quickly when needed. Upon pathogen detection, ECs can adopt an activated phenotype by upregulating adhesion molecules and chemokines to coordinate immune cell trafficking (Shen et al, 2010; Vandenbroucke et al, 2008). During infection, ECs exhibit semi-professional antigen-presenting properties and can directly engage and activate CD4^+^ and CD8^+^ effector memory T cells (Amersfoort et al, 2022). M2 macrophages surrounding postcapillary ECs secrete CXCL2 to recruit dermal leukocytes and promote the formation of inducible skin-associated lymphoid tissue, within which effector T cells are activated (Honda and Kabashima, 2016). In addition, ECs regulate immune responses by modulating IgG homeostasis and presenting tissue-specific homing signals (eg, VCAM-1 and E-selectin), thereby orchestrating immune cell trafficking to infection sites (Medrano-Bosch et al, 2023).

In homeostatic tissues, ECs represent a long-lived and quiescent population whose sustained barrier integrity and regulatory functions are essential for vascular homeostasis. To accommodate the diverse and dynamic requirements of the tissues, ECs exhibit substantial phenotypic plasticity, which enables context-dependent modulation of functional properties and cellular identity. This adaptability gives rise to pronounced structural and molecular heterogeneity across distinct vascular beds, underpinning tissue-specific vascular functions and responses to physiological stress or injury (Gurevich et al, 2021; Potente and Mäkinen, 2017). Single-cell RNA sequencing studies have shown that dermal ECs exhibit distinctive traits compared to ECs from other organs (Li et al, 2021). For example, immune-related genes (eg, CD74 and ACKR1) were enriched in dermal ECs. In addition, other genes related to angiogenesis (eg, S100A6) were also selectively expressed in dermal ECs (Lerchenmüller et al, 2016; Li et al, 2021). Overall, skin microvascular endothelium forms the front line of the skin’s interactions with the circulation and immune system. The unique molecular signatures of ECs help to sense injury and orchestrate wound repair through dynamic changes in phenotype.

## Endothelial Functions Across Different Phases of Wound Healing

### Hemostasis and coagulation

Immediately after skin injury, neurogenic reflexes and locally released vasoactive mediators induce vasoconstriction and limit blood loss (Figure 2a). The exposed collagen and tissue factor in the subendothelial matrix trigger platelet adhesion and activation, forming a platelet plug (Olgasi et al, 2024). ECs play a central role in maintaining hemostasis through the production of coagulation factors such as von Willebrand factor (Nightingale and Cutler, 2013), or by modulating vascular tone through nitric oxide-mediated vasodilation and vasoconstriction, as well as by regulating vascular permeability to preserve barrier integrity (Bazzoni and Dejana, 2004; Minshall and Malik, 2006). Following vascular injury, loss of endothelial barrier integrity exposes the subendothelial matrix, including collagen and von Willebrand factor, promoting platelet adhesion and activation through platelet receptors such as GPIb and integrin αIIbβ3 (Shen et al, 2026). At the same time, tissue factor expressed by activated endothelial and stromal cells initiates the extrinsic coagulation pathway by binding factor VII/VIIa, resulting in thrombin generation (Neubauer and Zieger, 2022). Thrombin then converts fibrinogen into fibrin and enhances platelet activation, leading to clot formation and prevention of blood loss (Neubauer and Zieger, 2022; Shen et al, 2026).

**Figure 2:**
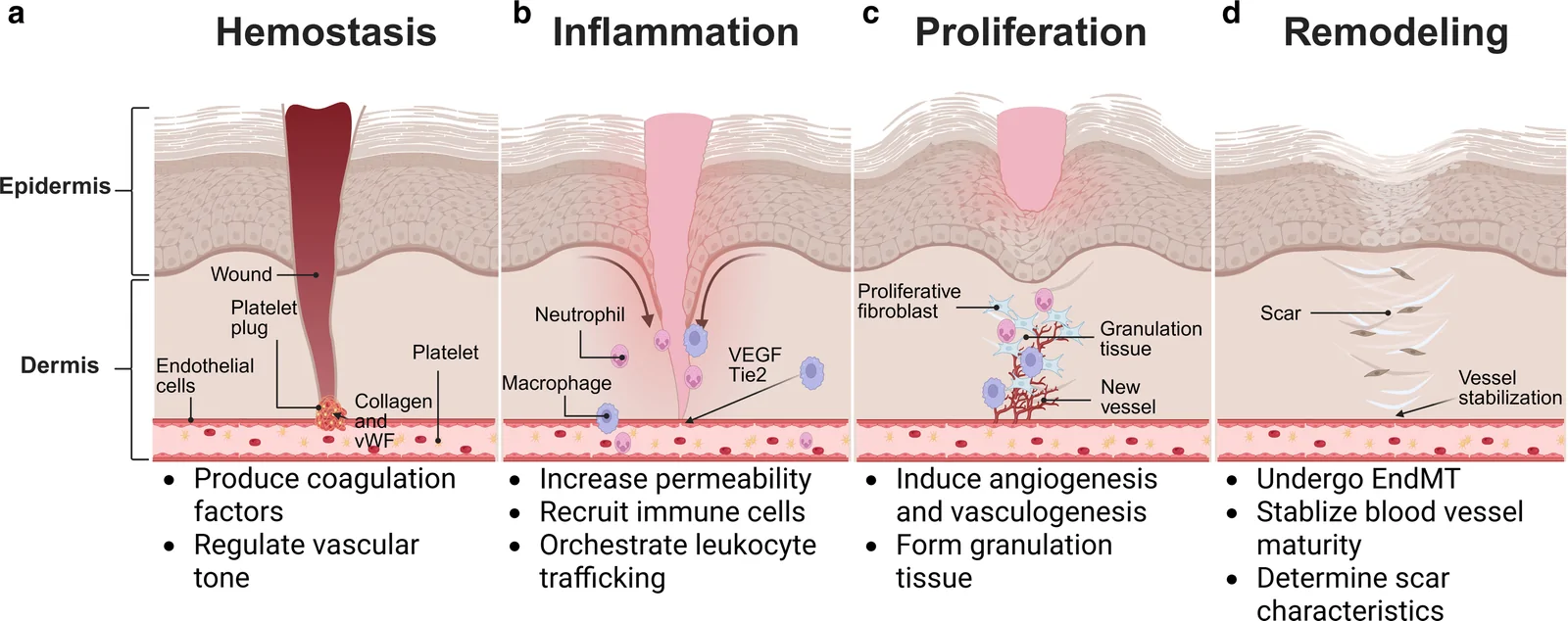
**Schematic figure illustrating the different phases of wound healing and roles of endothelial cells in each phase.** Wound healing normally consists of 4 sequential but also overlapping phases, **(a)** hemostasis, **(b)** inflammation, **(c)** proliferation and **(d)** remodeling. Created in BioRender. Li (2026) https://BioRender.com/brzjlw5. EndMT, endothelial to mesenchymal transition; Tie2, tunica intima endothelial kinase 2; VEGF, vascular endothelial growth factor; vWF, Von Willebrand Factor.

### Inflammation and immune cell recruitment

Within minutes to hours after wounding, an inflammatory response is initiated (Figure 2b). Activated ECs produce PGs (via COX-1) and nitric oxide, causing vasodilation and increased blood flow into the wound bed (Pober and Sessa, 2007; Schwentker et al, 2002). Junctional proteins (occludin and VE-cadherin) are loosened through actin-mediated contraction, leading to increased endothelial permeability (Rho et al, 2017; Schulte et al, 2011). As a result, plasma proteins and immune cells (neutrophils and monocytes) extravasate into the tissue (Pober and Sessa, 2007).

ECs are key regulators of inflammation and tissue repair. They serve as the primary interface for immune cell recruitment and trafficking, while actively modulating inflammatory responses through context-dependent cytokine production (Lallo and Bracaglia, 2024). ECs present antigens and adhesion proteins that selectively orchestrate leukocyte trafficking at each healing stage (Amersfoort et al, 2022). Neutrophils are the first responders, followed by macrophages (Rodero et al, 2013; Rodero et al, 2014). During the inflammatory phase, ECs are activated by damage-associated molecular patterns, hypoxic signals, and pro-inflammatory cytokines (Assis et al, 2026). After activation, ECs induce the expression of adhesion molecules (eg, ICAM-1 and VCAM-1) and secrete chemokines (eg, IL-8), leading to the sequential rolling, firm adhesion, and trans-endothelial migration of neutrophils and circulating monocytes into the wound bed (Assis et al, 2026; Zhang et al, 2021). As the inflammatory response progresses, infiltrating monocytes differentiate into macrophages that initially adopt a pro-inflammatory phenotype (Wynn and Vannella, 2016). These macrophages produce cytokines, including TNF-α and IL-1β, which further activate ECs and promote the recruitment of additional immune cells (Assis et al, 2026; Wynn and Vannella, 2016). Likewise, vascular ECs undergo dynamic phenotypic reprogramming, enabling them to regulate inflammatory responses (DiStasi and Ley, 2009). This interplay highlights that ECs not only permit leukocyte passage but also actively coordinate inflammation through bidirectional barrier signaling.

As inflammation resolves, macrophages undergo a phenotype transition from a pro-inflammatory state (M1) to an alternatively activated (M2) phenotype that supports angiogenesis (Galli et al, 2011; Rodero et al, 2013). These pro-healing phenotype macrophages often express VEGF and Tie2, which prepare the wound site for subsequent tissue formation (Fantin et al, 2010; Krzyszczyk et al, 2018; Willenborg et al, 2012). ECs in the local microenvironment begin to proliferate in response to these regenerative signals. However, sustained EC activation, as seen in chronic wounds, can exacerbate inflammation and edema (Schilrreff and Alexiev, 2022). Proper attenuation of the endothelial “inflammatory” phenotype is therefore as important as its initial activation (Sim et al, 2022).

### Proliferation and angiogenesis

During the proliferative phase of wound healing, granulation tissue is formed through the deposition of new connective tissue, occurring in parallel with coordinated processes such as reepithelialization, neovascularization, and immune modulation (Wallace et al, 2025) (Figure 2c). Hypoxia and factors released by macrophages and keratinocytes (eg, VEGF and FGF) activate local dermal ECs to proliferate and sprout new capillaries (Tejedor et al, 2024; Tonnesen et al, 2000). Early in the proliferative stage, blood vessels are primitive and leaky, where EC tip cells lead sprouts toward pro-angiogenic gradients, while stalk cells elongate behind. Notably, VEGF-A gradients and Notch signaling determine tip versus stalk cell identity, where tip cells exhibit high VEGF signaling activity and low Notch signaling activity, while stalk cells adopt the opposite signaling profile (Mack et al, 2017; Mamun et al, 2024). The new capillary loops formed through angiogenesis supply oxygen and nutrients essential for granulation tissue formation by supporting fibroblast activity and collagen deposition.

In parallel to angiogenesis, another mode of new vessel formation also occurs. This is called vasculogenesis, which is the de novo formation of blood vessels from endothelial progenitor cells (EPCs) during the prenatal and postnatal stages (Asahara et al, 1999; Flamme et al, 1997). The endovascular progenitor cells, surrogate of human EPCs in mice, are vessel-resident endothelial progenitor populations that contain self-renewal ability and give rise to transit-amplifying and definitively differentiated ECs during wound healing (Patel et al, 2017; Shafiee et al, 2018). The self-renewal capacity and differentiation of EPCs are regulated by NOTCH signaling and reactivation of SOX18, which contribute to driving vasculogenesis (Patel et al, 2017; Patel et al, 2016). In addition, lineage-tracing and transplantation experiments indicate that EPCs are vascular-resident rather than bone marrow-derived (Okuno et al, 2011; Patel et al, 2017).

As neovascularization proceeds, resident fibroblasts proliferate and infiltrate the provisional clot, where a subset differentiates into myofibroblasts that generate tensile forces to draw the wound edges together (Abdelhakim and Ogawa, 2025). The remaining fibroblasts deposit and organize the extracellular matrix (ECM) that stabilizes granulation tissue and guides subsequent tissue remodeling (Tracy et al, 2016). Granulation tissue consists of collagen bundles, macrophages, granulocytes, and abundant newly formed blood vessels (Landén et al, 2016).

Pharmacologically, this phase is targeted by pro-angiogenic therapies. In clinical trials, the administration of exogenous VEGF or FGF has been used to promote neovascularization with variable outcomes (Barrientos et al, 2014; Peeters et al, 2024).

### Remodeling and maturation

Once a robust and well-vascularized granulation tissue is established, the wound progresses to the remodeling phase, during which the ECM is restructured and strengthened, and excess blood vessels regress toward physiological vascular density (Figure 2d). Fibroblasts deposit Collagen type I, which replaces Collagen III, to provide tensile strength and structural integrity in the healed tissue (Gurtner et al, 2008; Smith et al, 2019).

Mechanical contraction of the wound also affects ECs. Increased matrix stiffness promotes fibroblast activation and myofibroblast differentiation through YAP/TAZ and TGF-β signaling, resulting in excessive ECM deposition and greater tissue contractility (Chu and Quan, 2024; Mierke, 2024). As the matrix stiffens, endothelial mechanosensing is altered, contributing to abnormal angiogenesis and endothelial dysfunction (Lim and Harraz, 2024). Increased tissue stiffness also enhances endothelial expression of adhesion molecules and inflammatory mediators, facilitating leukocyte recruitment and prolonging the inflammatory response (Chen et al, 2019; Mierke, 2024). In turn, persistent inflammation drives further ECM remodeling and tissue stiffening, creating a self-perpetuating cycle that contributes to hypertrophic scar formation and fibrosis (Chu and Quan, 2024; Mascharak et al, 2024). As myofibroblasts deposit and contract collagen, tissue perfusion decreases, and intravascular shear stress changes. Collagen compression can occlude some microvessels, leading to vascular regression (Bus et al, 2024). Recent studies note that as remodeling proceeds, pro-angiogenic signals (VEGF and FGF) diminish in the wound environment, which promote vessel closure (Huang K et al, 2025). The remaining capillaries remodel into more normal dermal capillaries, while many small vessels regress through apoptosis (Huang K et al, 2025; Wietecha et al, 2013).

Concurrently, excess ECs may undergo mesenchymal transition, leading to the selective remodeling of the temporary capillary plexus. Endothelial-to-mesenchymal transition (EndMT) is a process where ECs lose their endothelial identity and acquire mesenchymal characteristics, enhancing migratory and ECM-producing capacity, and fibrotic remodeling. During wound healing, this process contributes to myofibroblast accumulation and ECM deposition, leading to increased fibrosis and scar formation (Patel et al, 2018). Conditional silencing of Recombination Signal Binding Protein for Immunoglobulin Kappa J Region in mice, the downstream transcriptional effector of canonical NOTCH signaling, was found to accelerate EndMT and cause excessive scar formation (Patel et al, 2018), leading to an increase in *Sox9* expression in ECs (Zhao et al, 2021). In contrast, endothelial-specific deletion of *Sox9* reduced EndMT and resulted in smaller scar areas in mouse skin wound models (Zhao et al, 2021). Indeed, EndMT is accompanied by a large reprogramming of the EC transcriptome with chromatin opening at mesenchymal genes without closure of chromatin at endothelial genes. Inhibition of *Sox9* largely restores this mesenchymal transition, reducing mesenchymal gene expression and restoring endothelial gene levels (Zhao et al, 2024). Transient EndMT is thought to support tissue repair by facilitating vascular remodeling. However, sustained or excessive EndMT can lead to endothelial dysfunction, fibrosis, and pathological tissue remodeling (Xu and Kovacic, 2023). Recent studies have also identified SOX9 as an important regulator of endothelial cell fate (Fuglerud et al, 2022; Vu et al, 2023). By modulating chromatin accessibility and activating transcriptional programs associated with mesenchymal differentiation, SOX9 promotes endothelial phenotypic switching and may play a central role in regulating cell-state transitions (Fuglerud et al, 2022). The interaction between Notch signaling and SOX9 therefore represents a key regulatory axis governing endothelial identity and plasticity, although the precise molecular hierarchy remains incompletely understood (Vu et al, 2023; Xu and Kovacic, 2023).

In the remodeling phase and following neo-vasculature regression, blood flow returns toward normal dermal levels (Han et al, 2022; Hosseini et al, 2022b). During this phase, EC undergoes apoptosis or mesenchymal transition, and mature blood vessels stabilize through increased pericyte coverage and basement membrane deposition (Rodrigues et al, 2019).

In parallel, during the remodeling phase, lymphatic ECs undergo lymphangiogenesis, which is regulated by VEGF-C and VEGF-D, forming new lymphatic networks that facilitate the clearance of interstitial fluid and immune cells into lymphatic vessels (Martínez-Corral et al, 2012; Tammela and Alitalo, 2010). The lymphatic vessel wall becomes thinner and irregular, with lymphatic ECs extending into the lumen and surrounding matrix, leading to the extension of lymphatic vessels into the superficial dermis (Ji et al, 2004; Jian et al, 2024). Although the mechanisms of lymphatic vessel remodeling are not fully understood, impaired EC function has been found to disrupt the remodeling phase, leading to delayed wound healing and sustained inflammation (Brunner et al, 2023; Pollack et al, 2025).

The remodeling phase also determines scar characteristics. A mature scar exhibits a markedly reduced quantity of blood vessels compared to granulation tissue. In established scar tissue, the microvasculature resembles that of normal dermis, which primarily consists of a capillary network surrounded by dense collagen (Bus et al, 2024). Excessive remodeling, characterized by heightened EC apoptosis, can lead to tissue hypoxia and fibrotic stiffening, while underremodeling correlates with aberrant vascular density, impaired tissue maturation, and chronic inflammation. For example, hypertrophic scars exhibit increased microvessel density, immature capillaries, and sustained angiogenesis (Hosseini et al, 2022a; Johnson and DiPietro, 2013; Tan et al, 2024). Conversely, if ECs prematurely regress, as may occur in chronic wounds or diabetic ulcers, the tissue fails to fully revascularize and displays poor healing (Kolluru et al, 2012).

Physiological wound healing depends on well-coordinated angiogenesis and vascular remodeling. However, persistent endothelial activation has been implicated in the development of pathological scars, including hypertrophic scars and keloids (Fang et al, 2025; Wen et al, 2025). ECs contribute to the secretion of angiocrine factors, such as TGF-β, VEGF, platelet-derived growth factor (PDGF), and connective tissue growth factor, which stimulate fibroblast proliferation, myofibroblast differentiation, and ECM production, leading to the promotion of fibrosis (Wen et al, 2025; Zhang et al, 2020). In turn, activated fibroblasts remodel the ECM and increase tissue stiffness, which disrupts endothelial mechanosensing and contributes to abnormal angiogenesis and endothelial dysfunction (Eura et al, 2022). Endothelial dysfunction also increases vascular permeability, promotes leukocyte infiltration, and sustains inflammatory signaling, further impairing normal scar resolution (Fang et al, 2025; Wen et al, 2025). Together, these observations indicate that pathological scar formation is driven by ongoing interactions among ECs, fibroblasts, immune cells, and the ECM.

## Endothelial Dysfunction in Pathological Wound Healing

In diseases such as diabetes mellitus, peripheral arterial disease, chronic venous insufficiency, radiation, aging, and autoimmune disorders, the ECs’ behavior during wound healing can be disrupted (Rodrigues et al, 2019). In diabetes, ECs dysfunction is central to impaired skin wound healing. In chronic hyperglycemia, ECs undergo oxidative stress and glycation damage, leading to reduced proliferation, migration, and increased apoptosis (Yang et al, 2024). As ECs die or become senescent, angiogenic capacity declines and capillary perfusion suffers. Diabetic wounds often remain hypoxic and inflamed because new vessels fail to form or function properly. In addition, high glucose alters growth factor signaling. For example, VEGF signaling can become blunted by the upregulation of vascular endothelial growth factor receptor (VEGFR)1 decoy receptors, and nitric oxide (NO) production is reduced by endothelial nitric oxide synthase (eNOS) uncoupling. The net result is microvascular rarefaction and leaky, immature vessels (Boucher et al, 2017; Janaszak-Jasiecka et al, 2023).

A recent study found that in diabetic mice, endothelial casein kinase 2α (CK2α) was abnormally upregulated. The CK2α inhibitor was found to improve EC proliferation and rescue angiogenesis by restoring Hedgehog signaling in ECs (Zhu et al, 2023). In addition, endothelial-specific deficiency of Dab2 in diabetic mice led to reduced VEGFR2 signaling, poor angiogenesis, and delayed healing. In contrast, delivering Dab2 mRNA via endothelial-targeted nanoparticles restored Dab2 levels in ECs and rescued angiogenic function and wound closure (Bhattacharjee et al, 2025).

Aging is also associated with endothelial dysfunction and diminished vascular plasticity and adaptability, which contribute to poor skin repair. Aged skin shows reduced vascular density and delayed revascularization during wound repair. Cellular senescence is characterized by irreversible cell-cycle arrest accompanied by the development of a senescence-associated secretory phenotype (SASP) (Wilkinson and Hardman, 2022). In senescent ECs, the SASP includes pro-inflammatory mediators such as IL-1α/β, IL-6, IL-8, and TNF-α, which promote a persistent inflammatory microenvironment and induce senescence in neighboring endothelial, stromal, and immune cells through paracrine signaling (Bloom et al, 2023; Xie et al, 2026). These inflammatory factors also promote the recruitment and activation of neutrophils and monocytes, which sustain endothelial activation (Bloom et al, 2023). As a result, prolonged neutrophil infiltration leads to increased production of ROS, release of proteolytic enzymes, and formation of neutrophil extracellular traps, further damaging the endothelium and delaying the resolution of inflammation (Xie et al, 2026). Single-cell RNA sequencing results showed that wounds in aged mice subjected to chronic hyperglycemic stimulation exhibited markedly reduced wound healing efficiency and impaired angiogenesis compared with wounds in young mice. In aged mice, both dermal ECs and fibroblasts in wound sites show delayed regenerative capacity, characterized by decreased expression of angiogenic genes (eg, VEGF and angiopoietins) and increased expression of inflammation- and senescence-related genes (Li et al, 2025). Consistent with these findings, a recent study found that senescent dermal ECs secreted inflammatory factors that activated nociceptive neurons, which in turn triggered mast cell degranulation in aged mice. This ultimately caused dermal thinning and delayed healing (Wicaksono et al, 2025). Aging-related endothelial changes, including reduced proliferative capacity, basement membrane thickening, and chronic inflammatory signaling, are regarded as key contributors to delayed wound healing (Li et al, 2025; Scioli et al, 2014).

## Therapeutic Strategies Modulating EC Function

Given the centrality of ECs in healing, many therapies have been designed to modulate EC function in skin wounds. These strategies range from the direct administration of growth factors to sophisticated bioengineering approaches.

### Growth factor therapy

Topical or injectable VEGF, PDGF, and EGF have been used to enhance angiogenesis (Bai et al, 2018; Peeters et al, 2024; Weng et al, 2022) with VEGF-A being the most extensively studied mediator. Early trials delivered recombinant VEGF protein or plasmid DNA to chronic wounds, with variable outcomes (Yamakawa and Hayashida, 2019). More recently, mRNA-based VEGF-A has been used with promising outcomes. The VEGF-A mRNA (and modified FGF1 mRNA) was applied topically and resulted in increased angiogenesis and accelerated wound closure in diabetic mouse models (Tejedor et al, 2024). Although growth factors are regarded as powerful new therapies, single-factor administration strategies often have limited efficacy due to short half-lives and the complex microenvironment of wounds. Moreover, this therapy can cause unregulated angiogenesis, potentially leading to edema or aberrant vessel growth (Mullin et al, 2024). Finally, other approaches have attempted to regulate the EndMT through small interfering RNA inhibition of Sox9 to reduce scarring and accelerate healing (Zhao et al, 2021).

### Cell therapy and EPC transfer

Transplanting cells capable of vascular repair is an active area of clinical research. Autologous or allogeneic EPCs can contribute to regeneration by providing pro-angiogenic signals (Sim et al, 2019). Clinical studies have investigated the administration of bone marrow- or cord-blood-derived EPCs to improve chronic wound outcomes (Farabi et al, 2024). A novel approach is preconditioning EPCs (Shafiee et al, 2017), whereby exposure to a mesenchymal niche improves EPCs’ survival and regenerative capacity. Another recent study showed that treating EPCs with a TGF-β inhibitor before grafting yields enhanced EPCs survival in diabetic conditions (Su et al, 2025). When these EPCs were applied to diabetic mice, they significantly increased capillary density in addition to accelerating wound closure. Such preconditioning, or genetic enhancement of EPCs (eg, overexpressing pro-survival genes), is being actively investigated (Su et al, 2025). In addition, EPC-derived extracellular vesicles (EPC-EVs) can recapitulate these effects. A recent study found that EVs from human EPCs enhanced angiogenesis, increased circulating EPC mobilization, and accelerated wound healing in a diabetic mouse model (Bao et al, 2026). Another emerging approach is using induced pluripotent stem cell-derived ECs (or organoids) to create prevascularized grafts for application in large wounds (Gara et al, 2022; Yu et al, 2025; Zhang et al, 2024). Although currently at the experimental phase, these biofabricated vascular constructs hold significant potential for improving wound repair in severe burn injuries. Cell therapies offer powerful adjuncts for wound healing purposes. The transplanted cells may contribute to wound healing not only by differentiating into various skin cell types but also by secreting key growth factors that are able to promote tissue repair through enhanced endothelial activity. However, the efficacy of cell-based therapies in wound healing remains limited by high biomanufacturing costs, the potential immunogenicity of transplanted cells, and their limited survival after transplantation.

### Biomaterial scaffolds

Materials science-driven strategies are being applied to support ECs function during wound healing. Biomaterials that mimic the mechanical properties of native tissue have been shown to support endothelial homeostasis, promote organized neovascularization, reduce excessive inflammation, and improve wound repair (Shukla et al, 2026; Zhao et al, 2026). Advanced biomaterial constructs can be engineered to incorporate pro-angiogenic peptides, growth factors, or even living ECs to generate an instructive vascular template able to promote neovascularization (Hosseini et al, 2022b; Hosseini and Shafiee, 2023). For example, a peptide-modified collagen scaffold loaded with human ECs was used in a large burn wound model. This construct not only delivered viable ECs but also accelerated the recruitment of the host’s own ECs and neovessel formation. Such cell-seeded scaffolds integrate with the wound bed and promote granulation tissue formation (Song et al, 2023). Other bioengineering approaches include nanoparticles or microspheres that gradually release VEGF or PDGF to sustain angiogenic signaling in situ (Liu et al, 2023; Shi et al, 2018).

Several studies have highlighted repair strategies targeting ECs for improved wound healing outcomes (Fantini et al, 2024; Yao et al, 2026). Table 1 shows a summary of clinical trials targeting ECs to improve healing outcomes in non-healing skin wounds.

**Table 1:** A Summary of Clinical Trials Targeting Non-Healing Wounds Through Different Therapeutic Strategies Involving Endothelial Cells or Endothelial Progenitor Cells in Humans

Therapeutic Strategy	Target Pathway or Molecule or Cell Population	Clinical Context	Outcomes	References
**Growth Factor**	rhPDGF-BB (becaplermin) (FDA approved)	Chronic neuropathic diabetic ulcers (n = 382)	Increased complete healing rate and faster healing times	(Wieman et al, 1998)
	rhEGF	Chronic diabetic foot ulcers (n = 167)	Increased complete healing rate and faster healing times	(Park et al, 2018)
	PRP	Chronic diabetic foot ulcers (n = 150)	Improved wound healing	(Babaei et al, 2017)
	rhVEGF	Chronic diabetic neuropathic foot ulcers (n = 55)	Reduction in the ulcer size	(Hanft et al, 2008)
**Cell**	G-CSF-mobilized peripheral blood CD34^+^ cell	Chronic diabetic foot ulcers (n = 5)	Complete wound closure with increased vascular perfusion	(Tanaka et al, 2014)
	MNC-QQ cells (selectively increase of EPCs)	Chronic diabetic foot ulcers (n = 9, 10 wounds)	Reduced pain, and complete wound closure in 60% of patients	(Tanaka et al, 2022)
	PB-MNCs	Critical limb-threatening ischemia and diabetic ulcers	Higher healing rate	(Panunzi et al, 2022)
**Biomaterial Scaffolds**	Collagen/Gelatin Scaffold with bFGF	Chronic ulcers (n = 17)	Improved wound healing	(Morimoto et al, 2013)
	Fibrin-collagen hydrogel with stromal vascular fraction-derived endothelial cell population	Chronic diabetic foot ulcers (n = 10)	Improved wound healing	(Nilforoushzadeh et al, 2020)

Abbreviations: bFGF, fibroblast growth factor 2; EPCs, endothelial progenitor cells; FDA, food and drug administration; G-CSF, granulocyte colony-stimulating factor; MNC-QQ, mononuclear cell quality and quantity control culture; PB-MNCs, peripheral blood mononuclear cells; PRP, platelet-rich plasma; rhEGF, recombinant human epidermal growth factor; rhPDGF-BB, recombinant human platelet-derived growth factor-BB; rhVEGF, recombinant human vascular endothelial growth factor.

### Conclusion

Cutaneous ECs function as dynamic regulators at every stage of wound healing. They contribute to developing new blood vessels, regulating immune cell influx, and engaging in reciprocal crosstalk with fibroblasts and keratinocytes to promote tissue regeneration. Recent studies have investigated the unique characteristics of cutaneous ECs and identified skin endothelial subpopulations and distinct molecular signatures that regulate tissue angiogenesis, immune cell trafficking, and coordinated dermal regeneration during wound healing. ECs dysfunction underlies delayed healing or pathological scars, thereby positioning ECs as central determinants of wound outcome. Consequently, emerging therapeutic strategies increasingly aim to regulate endothelial activities through growth factors and gene delivery or bioengineering strategies designed to improve wound healing outcomes. Collectively, a comprehensive understanding of skin endothelial biology is essential to design next-generation therapeutics aimed at improving wound healing and reducing scarring.

## Ethics Statement

Not applicable.

## Data Availability Statement

All data supporting this study are included within the article.

## ORCIDs

Haiming Li: http://orcid.org/0000-0002-6991-8114

Laura Sormani Le Bourhis: http://orcid.org/0000-0002-0838-2088

Seen Ling Sim: http://orcid.org/0000-0001-6981-0317

Abbas Shafiee: http://orcid.org/0000-0002-8885-9025

Kiarash Khosrotehrani: http://orcid.org/0000-0002-6406-4076

## Conflict of Interest

The authors state no conflict of interest.
